# Supplementary material for: Patterns of Intron Gain and Loss in Fungi
Source: PLoS Biol. 2004 Nov 30;2(12):e422. doi: 10.1371/journal.pbio.0020422 (PMC532390; doi:10.1371/journal.pbio.0020422)
Supplement: Table S1 — Also available at http://genes.mit.edu/NielsenEtAl/. (4.3 MB ZIP). [file pbio.0020422.st001.zip › NielsenEtAl/html/664.html]

AN8721.1.NCU01980.1.MG03717.1.FG08879.1


```
 CLUSTAL W (1.82) Multiple Sequence Alignments - Introns Inserted


Sequence 1: NCU01980.1	515 aa
Sequence 2: FG08879.1	512 aa
Sequence 3: MG03717.1	454 aa
Sequence 4: AN8721.1	606 aa
Alignment Length: 634 aa
Number Identitical Residues: 118 aa
Alignment Score (without introns) 7266


MG03717.1 	----------~-------------------------------------------------
NCU01980.1	MECETDRAGC1MPKFTPEELAVNAVPVIRQATSG---------DAEPDWRPLFRHECTFN
FG08879.1 	MENRKKAP--~---FEPEEYPPGSLPLLHEQCG------------EASWIPMCRHACTFE
AN8721.1  	MARALALSRS~VQVCKSRLFTVASKSAITKRCLSNPIQYRSVSLFATTYAPSKDLNSRLH
          	       . .      .   .  : .   .   ... .  : :   .   .     .   

MG03717.1 	-~------MVNAVKNPNLLSSWLFRAEILSE---------------------------IE
NCU01980.1	P~VIFQDVMLNLIKNPNINSSWLFRADILYD---------------------------TE
FG08879.1 	T~RHFDEIMLNLVENPNLNSKWLFRADVIHD---------------------------ES
AN8721.1  	S1EHIGDTALYLLLNPGLTSSVLFRADILAQSGKCPTLAALEAQKQQETEEASTATAMTM
          	.     .  :  : **.: *. ****::: ::....: :: .:.....:..::::::   

MG03717.1 	HDQAEVCPVQGES--TLPRCPALQGFRLERAYLRQIIPRNTRRDSPMIQSCTFYVDS---
NCU01980.1	KNIQNGLETPLSELGDVPDIPSFDGFGLHRRLIRRLIPRSTMRDKPMDQTCLIYQSK-GS
FG08879.1 	SHSPVPADVKITE--DSPRTRDFEGFSRQRTMVRRLIPRNTLRDAPLDQTCAFYHSRPGA
AN8721.1  	AQNVIEEEKEVKEPVVEIPAREVPGFELHKTLIRRLIPRNQQLDMPVDQTCHLYSHAGAV
          	 .          .         . **  .:  :*::***.   * *: *:* :*    . 

MG03717.1 	---AQDPDTGIRRS-------LVLYLPHLDSTEEFPYYHPRVRGVGLLHEWNDE------
NCU01980.1	---DSEG--GLQRH-------LVIYLPHVSTEAEMPYYHPTVRGIAFHHEWSPT------
FG08879.1 	GELDSGGQTDLMRS-------LVLYMPHASSAGKLPFYHPKVKGICQLHEWNQS------
AN8721.1  	EQIEKEKELGIPHSRAPKERFMVIYTPHVSSKEELPYYHPLVRSMAFVYEFGYTGEFEGP
          	 .  .  . .: :  :...  :*:* ** .:  ::*:*** *:.:   :*:.  .. ...

MG03717.1 	---------QGKGTFSIHYQLFPEGSMTP-----RLQSTALNMLSILHKLGQGQLNGYVK
NCU01980.1	---------ETRGSVSVSYLYFEGGERPE-----KLTRIASHLLQAVYKHGQGRADGYQK
FG08879.1 	---------TEIGTVSVHFLLFDGEEDGEEVDQVKLGRIAYHLLQTIHKHGHGTAAGYIK
AN8721.1  	ENSIPRIAENPKGTMSIHFLPYENDVDSIS---ARLERSLTKLIEVQIRTTKGRLDPCRP
          	..: .  :.   *:.*: :  :       .    :*     :::.   :  :*       

MG03717.1 	RVH------HDRLVPKSALQDRYSGLRDRYAKELVGGWAESTDPMKHVFEDLCIAAFLIE
NCU01980.1	RVH------HDLLVPQDRVQNTYTALKQKYARALIESWVESTDPTKHVFEDLCIAAFLIE
FG08879.1 	RVN------HDVVVPRERFQDKYSELKNKYARSLVQSWLETTDPAKHVFEDLGIAAFLIE
AN8721.1  	STSSPYALIKDNVIPRNRVQDTYSRLKNKYAANLNERWIESTEPSKHVFEDLSIAAFLIE
          	 . :. :  :* ::*:. .*: *: *:::**  *   * *:*:* ******* *******

MG03717.1 	LWAEMYKTL--------------------EFPGFVDIGAGNGLLVHILNLEGYR2GWGFD
NCU01980.1	LWTDIYGRN--------------------FFPGFVDIGCGNGLLVHILNLEGFK~GWGFD
FG08879.1 	LWRDMYRGV--------------------DFPGFVDIGCGNGLLVHILKLEGYD~GWGFD
AN8721.1  	LWRDLYGAVPGDEREQQKQQSSTSKVGSGQFPGFVDIACGNGVLVYILISEGYS~GWGFD
          	** ::*   .... ......::::. .:. *******..***:**:**  **:  *****

MG03717.1 	ARARKSWHVYNGLQADG---QPTLQELVLIPSLVGQEVSPGKNDVEVSPQT---------
NCU01980.1	ARSRKSWATYSTRLETPTGVQESLQQLVLLPPPVSRAGISEISSEGFREE----------
FG08879.1 	ARERKSWSQYNSSFSGSP-SGKSLQRRLLLPSIIP-VETPDANTKVIDPK----------
AN8721.1  	ARRRKTWSIFPTDVQERLKEEIYIPKPFMDVLAAQNQGPIQNKNQDASMQSPLSEPQSGQ
          	** **:*  :             : . .:             .      ::. :...:..

MG03717.1 	---------VGVHNGVFPKGTFIISNHADELTPWTPLIATAS----GCPFLAIPCCSHNL
NCU01980.1	----------LVHDGRFPKGTFIISNHADELTPWTPILAAIS----DCPFIMIPCCSHDL
FG08879.1 	----------DVHDGTFPKGTFIISNHADELTPWTPILAAIS----QCPFISIPCCSHNL
AN8721.1  	SNGPNSGTSTSTSLSNLPKDTFIISNHADELTLWTPILSTLLNPANPPPFLAIPCCSHSL
          	:....:.::  .  . :**.************ ***::::  ..:.  **: ******.*

MG03717.1 	TGARFRAPRPTDKSKSHSAYSSLVAWVSDIATDCGWEVESEMLRIPSTRNTAIVGRHRTV
NCU01980.1	TGARFRAPAPKDKKKANSAYSSLVSWVTQITADCGWVAEQEMLRIPSTRNTAIVGRKRQG
FG08879.1 	TGDRWRAPPPRDKSKPKSMFASLVDWVAQIAEDCGWDVETEMLRIPSTRNTGLVGRRNTK
AN8721.1  	SGARHRFRPQSARPSATQNPQKQQGENGTHSQDE----EGSSEDMKERKGEEKEKNPETG
          	:* * *      : .. .   .        : *     * .   : . :.     . .  

MG03717.1 	DITEVAIDQVIARYGGSGDYCENAMKLVKSISPRRH-
NCU01980.1	DVSAIDIGVIVDRYGGTAGYLETVVKLVKSTNLDEKH
FG08879.1 	GAEEIDMHAVLQKYGGVQGYYSNVAKLLKSTPRGH--
AN8721.1  	DLEQMRKDKLAAQNPH---------------------
          	.   :    :  :
```
